# The influence of formative assessment on academic performance: exploring the role of teachers’ emotional support

**DOI:** 10.3389/fpsyg.2025.1567615

**Published:** 2025-04-08

**Authors:** Junsheng Wu, Xin Yu

**Affiliations:** ^1^Department of Curriculum and Instruction, The Education University of Hong Kong, Tai Po, Hong Kong SAR, China; ^2^School of Materials and New Energy Xingzhi College, South China Normal University, Shanwei, China

**Keywords:** formative assessment, academic performance, teachers’ emotional support, mediation, structural equation modeling (SEM)

## Abstract

Formative assessment is widely recognised as a vital tool for improving student performance. However, how formative assessment effectively influences academic performance remains unclear. This study explores the mediating role of emotional support of teachers in this association. By reviewing the recent literature, we explore how formative assessment can indirectly influence student performance through the emotional climate created by teachers. The study was analyzed using a structural equation model to explore the impact of formative assessment on the academic performance of 280 middle-school students in the South Chinese region. The results showed a significant positive relationship between formative assessment and teachers’ emotional support, as well as a significant positive relationship between teachers’ emotional support and academic performance. Furthermore, we also confirmed that teachers’ emotional support plays a mediating role in the relationship between formative assessment and students’ academic performance. These implications illuminate the importance of integrating emotional support strategies into formative assessment to improve educational outcomes. Several limitations of this study are discussed (e.g., cross-sectional design, cultural constraints, and reliance on self-reported data).

## Introduction

Formative assessment is usually continuous and feedback-oriented, and is an integral part of the educational process. Many studies have confirmed its effectiveness in promoting academic performance (Hattie and Timperley, 2007; McCallum and Milner, 2020; Morris et al., 2021); however, some researchers have raised doubts about its effectiveness (e.g., Bennett, 2011; Briggs et al., 2012; Boström and Palm, 2023), indicating that its underlying mechanisms remain unclear. There is still some controversy regarding how formative assessment affects student academic achievement, particularly concerning potential mediating or moderating factors, which require further exploration. The mechanisms through which formative assessment affects student achievement are multifaceted and require further investigation. One potential mediating factor was teachers’ emotional support. Teachers’ emotional support, including positive interactions, encouragement, and a nurturing classroom environment, has been shown to significantly affect students’ engagement, motivation and academic achievement (Jennings and Greenberg, 2009; Reyes et al., 2012; Romano et al., 2021; Yang et al., 2021). However, empirical research on how formative assessment influences academic performance through teachers’ emotional support remains limited.

This study aims to fill this research gap by exploring the interaction between formative assessment and teachers’ emotional support and examining their combined effects on academic performance.

## Literature review

### Formative assessment and academic performance

Formative assessment involves the ongoing evaluation of student learning to provide feedback to guide instruction and improve student performance (Black and Wiliam, 1998). Wiliam (2011) outlined five core strategies for formative assessment including: (1) Ensuring that all participants have an explicit comprehension of the success criteria and learning goals by clarifying the intended learning outcomes. (2) Designing classroom activities, discussions, and tasks that encourage students to demonstrate their learning progress. (3) Offering feedback that drives learning improvement. 4. Fostering peer-assisted learning, utilizing students’ collective knowledge as a resource for teaching. 5. Guiding students to own their learning. Research has highlighted its positive effects on achievement. For instance, Xuan et al. (2022) confirmed that commonly formative assessment exerted a positive, although modest impact on reading attainment of students. Similarly, a meta-analysis led by Kingston and Nash (2011) found that formative assessment practices were associated with moderate-to-large increases in student achievement. Andersson and Palm (2016) explored the impact of Swedish fourth-grade mathematics teachers’ practices on student achievement after taking part in the professional development program on formative assessment. Their results showed that classes instructed by lecturers who received the intervention performed significantly well in comparison to the control group in the post-test. This indicates that improving teaching practices by integrating formative assessment strategies can effectively enhance student performance. Li (2016) used U.S. data from PISA 2009 and found, through structural equation model analysis, that formative assessment demonstrated both indirect and direct positive correlation with reading performance of students. The indirect relationship is mediated through teacher-student relationships and reading attitudes. Recent research by Lu and Cutumisu (2022) highlights the role of formative assessment in mediating the relationship between attendance and academic performance in a TEL-based course. The findings suggest that online formative assessment, along with online self-regulated learning, enhances performance by fostering engagement, indicating that mandatory attendance alone is insufficient for academic success. McCallum and Milner (2020) explored the effectiveness of formative e-assessments in first-year courses, highlighting positive student perceptions of their role in monitoring progress and enhancing learning. The findings also underscored the value of formative e-assessments in fostering student engagement and enabling early academic intervention. Overall, due to the rapid development of artificial intelligence, the forms of formative assessment seem to be changing.

However, a small number of studies indicate that formative assessment exhibited insignificant impact on academic achievement. For instance, Yin et al.’s (2008) experimental study showed that formative assessment was not significantly effective in improving students’ motivation, conceptual change, or scientific achievement. This outcome may be influenced by factors such as classroom management styles of instructors and the extent to which informal evaluation is utilized. But overall, the effectiveness of formative assessment practices was mentioned quite frequently. This suggests that, while there are conflicting results, general consensus supports the positive impact of formative assessment, and that differences across studies may be attributable to contextual factors or differences in implementation methods. Additionally, some studies may have failed to capture nuances in the implementation of formative assessment, such as the quality of feedback or the timing and frequency of assessment, all of which could affect its effectiveness. Therefore, while some studies show limited or no effects, they do not necessarily undermine the broader evidence supporting the value of formative assessment when applied in the right and appropriate context.

Consequently, the hypothesis H1 is postulated:

*H1*: Formative assessment is positively associated with academic performance.

### Teachers’ emotional support and academic performance

Emotional support belongs to social support, and teachers’ emotional support is characterized by providing students with love, trust, and compassion (House, 1981). Reportedly, emotional support positively influences students’ academic engagement (Shen et al., 2024). Social support theory advocates that emotional support establishs a sense of security (Drageset, 2021), and it can significantly improve students’ academic performance by increasing learning motivation and reducing stress by creating a supportive learning environment (Chen and Huang, 2024). Likewise, Self Determination Theory (SDT) suggests that (Ryan and Deci, 2020), if emotional support of teachers meets fundamental psychological needs of students (e.g., relatedness), it can stimulate their intrinsic motivation and promote their academic achievement and learning behaviors. For example, a study by Ruzek et al. (2016) found that tutors’ emotional support noticeably impacts motivation and engagement of their students, which are essential for academic achievement. Furthermore, Yang et al. (2021) found that instructors’ emotional support can positively affect the mathematics performance of boys and girls in primary and secondary schools. In addition, Kashy-Rosenbaum et al. (2018) also emphasized that the emotional support of class teachers is important for students’ academic performance. Research at the forefront of SDT theory has started to consider the integration of digital technology support. Chiu (2021) proposed a digital support design aimed at promoting student engagement in blended learning by satisfying the three needs (autonomy, competence, and relatedness) outlined in self-determination theory. The study found that, compared to teacher support, digital support was more effective in enhancing student engagement, with the relationship between digital support and student engagement varying depending on the types of support provided, learning expertise, and emotional design. A review by Li et al. (2024) pointed out that SDT-based interventions can help teachers design effective teaching strategies to meet the three basic needs of students in chatbot learning environments. By fulfilling these needs, teachers can motivate students with varying levels of ability, thereby addressing the digital divide caused by chatbots and promoting shifts in student motivation and learning outcomes.

Hypothesis H2 is presented as:

*H2*: Teachers’ emotional support is positively associated with academic performance.

### The mediating role of teachers’ emotional support

Creating an environment that fosters motivation and emotional support is essential for a child’s overall development (Garcia-Peinado, 2023). Attachment theory suggests that secure attachment leads to better emotional regulation and resilience (Bowlby, 1969). García-in their review, García-Rodríguez et al. (2023) explored the connection between parent–child attachment and teacher-student relationships, offering new insights into the application of teacher emotional support and attachment theory in education. The relationship between students and teachers is not only influenced by early parent–child attachment experiences, but also evolves into new attachment patterns through interactions with teachers during the school years. In other words, the formation of teacher-student relationships is not simply a continuation of early attachment, but may involve the development of distinct psychological representations (Schuengel, 2012). Overall, teacher-student attachment is influenced not only by the student’s early attachment experiences, but also by the teacher’s personal characteristics, which may be related to students’ academic performance, engagement, and learning processes (García-Rodríguez et al., 2023). Teachers, as highly influential individuals, often possess high emotional intelligence, enabling them to effectively use emotions to influence others (e.g., caring for students and respecting students) (Tripon, 2023). When teachers provide emotional support, they help students feel valued and understood, building secure relationships that increase students’ motivation to engage with formative feedback. Formative assessment can provide teachers with opportunities to demonstrate emotional support by providing constructive feedback and recognising students’ efforts. Supportive feedback augments emotional and academic resilience of students (Ruzek et al., 2016). Meanwhile, students are more inclined to involve in the feedback provided during formative assessment when they perceive their class teachers as emotionally supportive, leading to improved academic outcomes (Gikandi et al., 2011).

Hence, H3 and H4 are hypothesized that:

*H3*: Formative assessment has a significant positive association with teachers’ emotional support.

*H4*: Teachers’ emotional support mediates the relationship between formative assessment and academic performance.

## Methods

### Participants and procedure

This study employed a cross-sectional survey design using a convenience sampling method to investigate the research questions. The participants were selected based on availability and willingness to participate. Data was collected on-site through paper-based questionnaires distributed to students at the end of the semester. The surveys were conducted in classrooms during a scheduled class period, ensuring that participation did not interfere with students’ regular academic activities.

All participants voluntarily took part in the study, with no incentives offered for participation. Participation was entirely anonymous, and respondents were assured that their responses would be treated confidentially. Ethical considerations were taken into account, and informed consent was obtained from all participants and their parents before data collection. This study included 302 eighth-grade middle-school students from South China. After eliminating invalid questionnaires, 280 valid responses were obtained. The elimination criteria were: (1) more than 50% of the answers were missing and (2) all answers were consistent. The respondents’ ages ranged from 14 to 18 years (M = 16.15, SD = 0.29); 127 were female (45.36%; M = 16.13, SD = 0.49), and 153 were male (54.64%; M = 16.16, SD = 0.48). The overall missing data value was 0.52%, and since the data did not meet the assumption of multivariate normal distribution, we used the MICE package in R to perform multivariate imputation for the missing data (R Core Team, 2019).

## Instruments

### Formative assessment practice

This study adopts the validated Teacher Formative Assessment Practices Scale developed by Yan and Pastore (2022) to measure the formative assessment practice. The scale consists of 10 items in total, including two factors: ‘teacher-directed formative assessment (TdFA)’ (e.g., “I clarify what is valued for each assessment task”) and ‘student-directed formative assessment (SdFA)’ (e.g., ‘I ask students to provide feedback to help peers improve’). TdFA is used to measure teacher-guided formative assessment practices, while SdFA is used to measure student-guided formative assessment practices. The scale is ranked on a 6-point scale, ranging from 6 (very frequently) to 1 (never). Since this study focuses on students’ perceptions rather than teachers’ self-reported practices, the scale was adapted by modifying the subject of each item to ‘My teacher’ (e.g., ‘My teacher clarifies what is valued for each assessment task’). This adaptation ensures that the scale measures students’ perspectives on how teachers implement formative assessment strategies rather than teachers’ self-evaluations. Higher scores on this scale indicate that students perceive their teachers as using formative assessment practices more frequently.

In order to make simpler the model and gauge the main effects of the constructs, a 2nd-order CFA was conducted by merging the 2 sub-factors: TdFA (6 items, Cronbach *α* = 0.70) and SdFA (4 items, Cronbach α = 0.75).

### Perceived teacher emotional support

The emotional support subscale of the Teacher Support Scale, developed by Wu et al. (2024), was employed to measure students’ perceived teacher support of students. This subscale comprised 6 items (such as, ‘My teachers encourage me to study hard,’ Cronbach α = 0.933). Higher scores on this scale suggest that students perceive greater emotional support from their teachers.

### Academic performance

Physics final exam scores of students were selected as an academic performance indicator. At the end of each school year, an exam with unified content and scoring standards is organized. The final exam serves as a standardized assessment tool, ensuring consistency in content, scoring criteria, and examination format across all schools. Compared to assignments, classroom quizzes, or mid-term exams conducted during the semester, it provides a more objective and comprehensive evaluation of students’ overall mastery of the course. Moreover, the final exam covers the entire semester’s curriculum, allowing for an assessment of students’ holistic knowledge acquisition. In contrast, individual tests or assignments conducted during the semester may only evaluate specific topics, making them less effective in reflecting students’ overall academic performance. In addition, semester-based coursework grades may be influenced by teachers’ subjective evaluations, potentially introducing bias. A standardized final exam reduces such biases, ensuring that the assessment of students’ academic performance remains more objective and comparable across different schools. In the data analysis process, the physics scores of 8th-grade students from various schools were included. We use standardized test scores to evaluate students’ physics academic performance.

### Data analysis

SPSS software was used to calculate descriptive statistics, Cronbach’s *α*, Ordinal α, and correlation coefficients. CFA and SEM analyses were undertaken using R (R Core Team, 2019). The measurement and hypothesized models were then tested. As the data did not satisfy normal distribution, a robust maximum likelihood method was used. Although MLR is not specifically designed for ordinal variables and does not directly rely on polychoric correlation matrices for estimation, previous studies have suggested that it is appropriate to use MLR for factor analysis or SEM when the number of response categories per item is sufficiently large (e.g., five or more) (Raykov, 2012). This is because, when the response categories are numerous, treating these ordinal variables as continuous does not significantly increase the variability of parameter estimates, making this approach acceptable (Johnson and Creech, 1983). For the fit data index, we used the criteria of RMSEA and SRMR values lower than 0.08, and CFI and TLI values higher than 0.90 (McDonald and Ho, 2002). The indirect effect was estimated using a model with 5,000 resamples and percentile bootstrap confidence intervals. When the 95% confidence interval of the estimate did not have 0, the indirect effect was assumed statistically significant (Hayes, 2017).

## Results

### Preliminary analysis

Table 1 presents Cronbach’s *α*, Ordinal α and Spearman’s correlations for each variable. A significant positive association was found between formative assessment practices and teachers’ perceived emotional support (r = 0.55; r = 0.38). Moreover, a significant positive association was documented between perceived emotional support from teachers and academic performance (r = 0.29). In the same vein, a significant positive association was established between formative assessment practice and academic performance (r = 0.20; r = 0.16).

**Table 1:** Descriptive statistics and correlations.

	Cronbach’s *α*	Ordinal α	AP	TdFA	SdFA	PTES
AP	-	-	-			
TdFA	0.929	0.947	0.20^***^	-		
SdFA	0.869	0.900	0.16^**^	0.60^***^	-	
PTES	0.950	0.969	0.29^***^	0.55^***^	0.38^***^	-

Academic Performance: AP, Teacher-directed Formative Assessment TdFA, Student directed Formative Assessment: SdFA, Perceived Teacher Emotional Support: PTES. ***p < 0.001, **p < 0.01, *p < 0.05.

**Figure 1:**
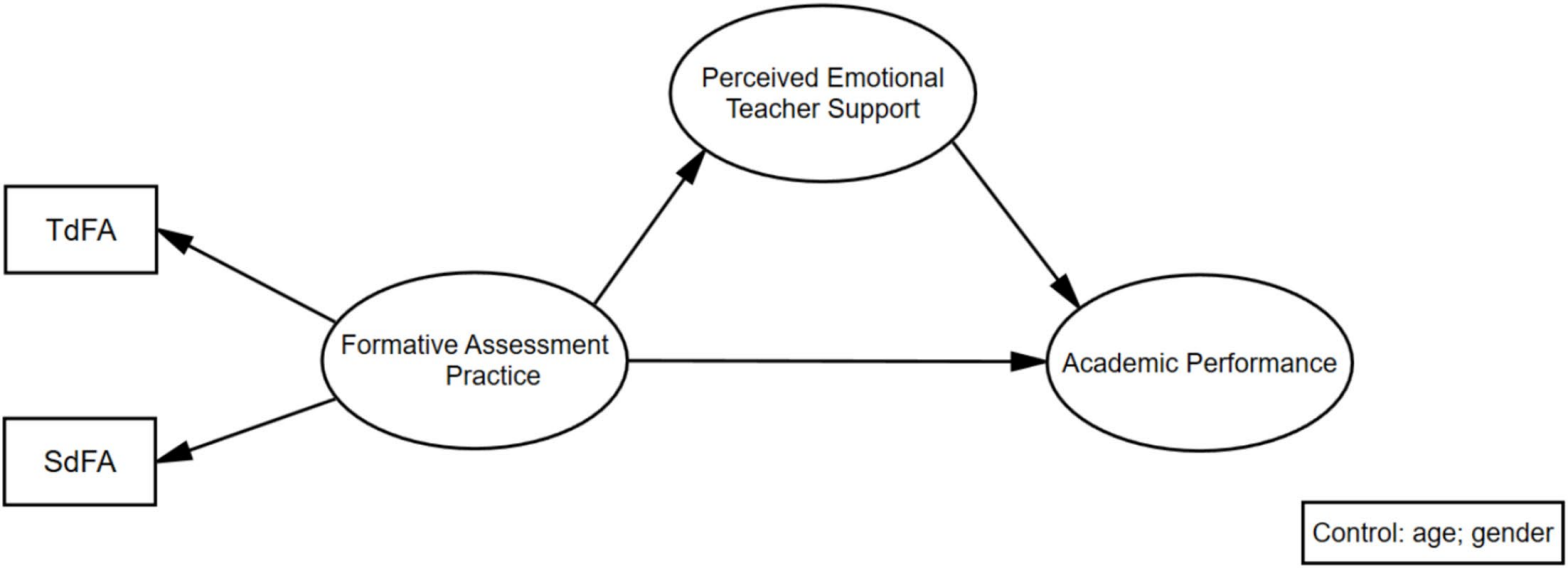
Hypothesis model.

To enhance the model fit in the CFA, the principle for deleting items is based on the theory of the scale as well as the opinions of the teachers. At the same time, following the criteria of Floyd and Widaman (1995), items with factor loadings below 0.4 are deleted. In this study, after deleting items #03 (My teacher uses various assessment activities in the classroom to check our mastery of course content.), #06 (My teacher provides suggestions for us to improve our performance.) and #07 (My teacher asks us to evaluate our peers’ work.), the SEM of the formative assessment practice scale obtained an acceptable model fit: S-Bχ^2^/df = 1.876, CFI = 0.987, TLI = 0.031, SRMR = 0.075, RMSEA = 0.071. All factor loadings are greater than 0.7. The average variance extracted (AVEs) of TdFA and SdFA are 0.77 and 0.71, respectively, both greater than 0.5 (Hair et al., 2017). The composite reliability (CRs) are 0.93 and 0.88, respectively, both greater than 0.7 (Hair et al., 2017). Similarly, to improve the CFA model fit, we deleted item #03 (My teacher encourages me to study hard.) according to the standard. As a result, the teachers’ emotional support scale showed a satisfactory model fit: S-Bχ2/ df = 1.447, CFI = 0.995, TLI = 0.991, SRMR = 0.013, RMSEA = 0.067. The hypothesised model (Figure 1) showed a good fit with the empirical data: S-Bχ2 / df = 1.078, CFI = 0.999, TLI = 0.996, RMSEA = 0.016, and SRMR = 0.026.

### Mediation analysis

**Figure 2:**
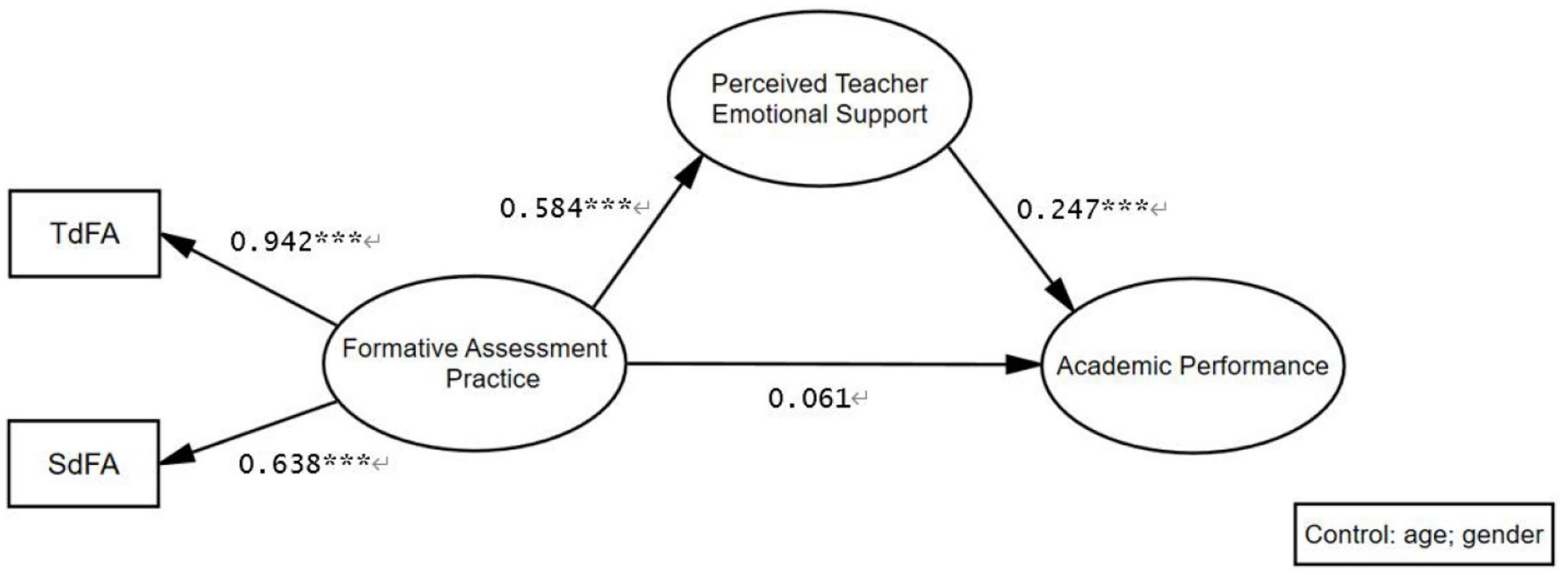
Model with standardized coefficients. *** *p* < 0.001, ** *p* < 0.01, * *p* < 0.05.

The results confirmed the following points (Figure 2): (1) Perceived teacher emotional support was positively associated with academic performance (*β* = 0.247, *p* < 0.001), which supports H2; (2) Formative assessment practices positively linked with perceived emotional support (β = 0.584, *p* < 0.001), which supports H3; (3) Formative assessment practices had no direct association with academic performance (β = 0.061, *p* > 0.5), which do not support H1. Lastly, a bias-corrected bootstrap sampling was utilized to ascertain whether the aforementioned mediation effect was statistically significant. Mediation analysis showed that teachers’ emotional support significantly mediated the association between formative assessment practices and academic performance. The indirect effect of teacher emotional support was significant (standardized indirect effect = 0.144, 95% CI [1.719, 6.359]), which supports H4. This indicates that the influence of formative assessment practices on grades can be explained by an increase in teachers’ emotional support.

## Discussion

In our first research hypothesis, we hypothesized that formative assessment practices would predict academic performance. However, consistent with the study by Yin et al. (2008), our results also showed that formative assessment practices did not significantly predict academic achievement. Although the effectiveness of formative assessment is frequently discussed (Bennett, 2011; Black and Wiliam, 1998; Dunn and Mulvenon, 2009), this is not always the case. One possible explanation is that students may not be in a supportive learning environment, which may affect their acceptance and utilization of formative assessment feedback. According to SDT (Ryan and Deci, 2020), when teachers provide positive emotional support, students are more likely to feel a sense of belonging, which may help them be more receptive to feedback and take proactive actions to improve their learning. A learning environment lacking emotional support may lead students to react negatively to feedback or even avoid it, thereby diminishing the effectiveness of formative assessment. The quality of the teacher-student relationship plays an imperative role in the effectiveness of formative assessment. Specifically, positive teacher-student affiliations, characterized by trust, respect, and emotional support, produce an atmosphere in which students are encouraged to take risks and make mistakes. When teachers make moderate efforts to express emotional support for students, students may be encouraged to establish positive interactions with teachers (Shen et al., 2024). When students perceive that their teachers support them, they are more likely to respond positively to formative assessment, thereby improving their academic performance. In classrooms with greater levels of teacher support, students report greater peer acceptance and classroom engagement compared to those in classrooms with less support (Hughes et al., 2005).

To further enhance the robustness of the research conclusions, future studies should explore potential confounding variables, such as students’ socioeconomic status (SES), previous academic achievement, personality traits, and classroom environment. These factors may be closely related to the effectiveness of emotional support and may influence students’ responses to formative assessment. For example, Sortkær (2019) found that high-SES students are more likely to view teacher feedback as an equal dialogue compared to low-SES students, which may also affect their perception of teacher emotional support. Additionally, students’ academic background, personality traits, or psychological state may moderate their acceptance of teacher support and how they use feedback. By controlling for or considering these factors, future research can provide a more comprehensive understanding of the complex relationship between emotional support and formative assessment, offering more precise guidance for optimizing educational practices.

## Implications for practice

This research adds to the understanding of how formative assessment can be optimized by integrating emotional support strategies. This has far-reaching implications for educational practice. These findings highlight the critical role teachers’ emotional support plays in improving the formative assessment’s effectiveness of student performance. This relationship emphasizes the holistic approach to education that integrates both cognitive and emotional dimensions. By focusing on the interaction between formative assessment and emotional support, we can develop more comprehensive strategies that not only improve academic achievement but also foster supportive learning environments.

Teachers should not only be trained in effective formative assessment techniques but also provide emotional support to students. Emotional support strategies can be incorporated into formative assessment practices using various methods. For example, teachers can use positive reinforcement to recognize students’ efforts and progress, thereby improving their self-esteem and motivation. In addition, creating potential prospects for students to reflect on their learning and express their emotions about their progress can help teachers provide targeted emotional support. In addition, peer learning can also be guided by teachers. Collaborative knowledge creation is an effective way for students to learn (Malan, 2020). Peer learning promotes cooperation through group work and peer review, fostering emotional investment in the process (Steenkamp and Brink, 2024). This dual approach helps create a supportive and conducive learning environment, ultimately improving student achievement.

## Limitation

Various limitations are also associated with this research. First, as a cross-sectional analysis, this study cannot predict causal associations between the variables. However, the findings from this study provide valuable insights for the design of future longitudinal and intervention studies. Future research could consider using longitudinal designs to better explore causal relationships or conduct intervention studies to assess the impact of specific factors. Second, the participants in this study are all Chinese. Most classrooms in contemporary East Asia are teacher-centred (Jiang et al., 2021), and a good teacher is often seen as someone who can strictly control the classroom process (Zhu et al., 2010). In contrast, when Western students perceive their teachers as stricter, their motivation and self-efficacy tend to decrease (Jiang et al., 2021). This difference may affect the cross-cultural applicability of the findings. Due to cultural differences, the effectiveness of the findings may need to be further explored in other countries. Additionally, the sample should be expanded to include greater diversity and representativeness, such as students from different grades (e.g., elementary and high school) and regions (e.g., northern and western China), to enhance the generalizability of the findings. Third, due to sampling limitations, the age range of the participants is restricted. A study involving a broader age range could increase the generalizability of the findings. Fourth, all the employed scales are self-report estimates, which may introduce common method bias. Future research could address this by incorporating multiple data sources, such as teacher evaluations, peer assessments, or classroom observations, to provide a more comprehensive measure of academic performance and emotional support. Response bias, as participants might answer in socially desirable ways or misinterpret questions. Lastly, academic performance was measured solely through physics exam scores, which may not capture a full range of learning outcomes. Future research should include multi-subject or competency-based metrics, such as problem-solving skills, to provide a more holistic assessment of student development.

## Conclusion

Formative assessment acts as a powerful instrument to improve student achievement. Nevertheless, its impact was greatly enhanced when combined with the teachers’ emotional support. This article highlights the value of a holistic method of education that simultaneously considers both cognitive and affective characteristics. Future research should explore this relationship and develop strategies to implement these findings in classroom settings.

## Data Availability

The datasets presented in this article are not readily available because they are owned by the collaborating school, which has imposed confidentiality restrictions to protect the privacy of participating students. Requests to access the datasets should be directed to corresponding author, via email: s1143409@s.eduhk.hk.
